# A Neural Signature Encoding Decisions under Perceptual Ambiguity

**DOI:** 10.1523/ENEURO.0235-17.2017

**Published:** 2017-11-21

**Authors:** Sai Sun, Rongjun Yu, Shuo Wang

**Affiliations:** 1 School of Psychology, Center for Studies of Psychological Application, and Key Laboratory of Mental Health and Cognitive Science of Guangdong Province, South China Normal University, Guangzhou, 510631, People’s Republic of China; 2 Department of Psychology, National University of Singapore, Singapore, 117570; 3 Department of Chemical and Biomedical Engineering and Blanchette Rockefeller Neurosciences Institute, West Virginia University, Morgantown, WV 26506

**Keywords:** ambiguity, decision, late positive potential, stimulus-driven, task-driven

## Abstract

People often make perceptual decisions with ambiguous information, but it remains unclear whether the brain has a common neural substrate that encodes various forms of perceptual ambiguity. Here, we used three types of perceptually ambiguous stimuli as well as task instructions to examine the neural basis for both stimulus-driven and task-driven perceptual ambiguity. We identified a neural signature, the late positive potential (LPP), that encoded a general form of stimulus-driven perceptual ambiguity. In addition to stimulus-driven ambiguity, the LPP was also modulated by ambiguity in task instructions. To further specify the functional role of the LPP and elucidate the relationship between stimulus ambiguity, behavioral response, and the LPP, we employed regression models and found that the LPP was specifically associated with response latency and confidence rating, suggesting that the LPP encoded decisions under perceptual ambiguity. Finally, direct behavioral ratings of stimulus and task ambiguity confirmed our neurophysiological findings, which could not be attributed to differences in eye movements either. Together, our findings argue for a common neural signature that encodes decisions under perceptual ambiguity but is subject to the modulation of task ambiguity. Our results represent an essential first step toward a complete neural understanding of human perceptual decision making.

## Significance Statement

Humans have a dedicated neural system to make decisions in ambiguous situations. Neuroimaging and electrophysiological studies have revealed brain regions and neural signatures in coding perceptual ambiguity, but it remains unknown whether there exists a common neural substrate that encodes various forms of perceptual ambiguity. Here, we revealed a common neural signature, the LPP, that encoded decisions under perceptual ambiguity. Using task instructions with different levels of ambiguity, we further showed that this neural signature was modulated by task ambiguity. Our findings highlight a common neural substrate underlying perceptual decision-making under ambiguity.

## Introduction

Assessing decision ambiguity is critical for decision-making. Although many studies have focused on value-based decisions in the face of risk (uncertainty with known probabilities) and ambiguity (uncertainty with unknown probabilities due to missing information) and revealed a distributed network of brain areas in value-based decision-making under risk and ambiguity (Hsu et al., 2005; Krain et al., 2006), we often encounter perceptual decisions when the mapping of stimulus category to choice was ambiguous. In the perceptual domain, interpreting ambiguous stimuli engages brain areas including bilateral inferior frontal regions (Sterzer and Kleinschmidt, 2007). In particular, one highly salient stimulus category encountered in everyday life that features pronounced ambiguity are facial expressions of emotions, which are frequently confused with one another (Young et al., 1997). Ambiguous emotional faces relative to unambiguous emotional faces activate cingulate cortices (Simmons et al., 2006), and single neurons in the human amygdala signal levels of emotion ambiguity (Wang et al., 2017). However, it remains unknown whether various forms of perceptual ambiguity share the same neural substrate and whether the coding of perceptual ambiguity is further modulated by top-down signals.

Previous literature has focused on a scalp-evoked late positive potential (LPP, beginning ∼400 ms after stimulus onset) that is associated with evaluation of ambiguous information. The LPP is sensitive to stimulus uncertainty (Sutton et al., 1965) and differentiates ambiguous facial expressions (Calvo et al., 2013) and racially ambiguous faces (Willadsen-Jensen and Ito, 2006). Earlier notions that the LPP might be specialized in processing affective pictures (Cuthbert et al., 2000; Schupp et al., 2000, Leite et al., 2012) have been supplemented by accounts that the LPP has diverse functions in social and economic evaluation: the LPP is not only involved in evaluating socially relevant concepts (Cunningham et al., 2005) and modulated by social comparisons (Wu et al., 2012), it is also associated with evaluation of good versus bad behavior in moral judgments (Yoder and Decety, 2014) and encodes positive versus negative outcomes in economic decision-making (Hajcak et al., 2005). In addition, a recent line of research showed that the LPP plays a key role in forming decisions: the LPP is not only associated with accumulating sensory information, but also plays an important role in determining choices (O’Connell et al., 2012; Kelly and O’Connell, 2013; Murphy et al., 2015). In a recent study, we have shown that the LPP differentiates levels of ambiguity and is specifically associated with behavioral decisions about choices that are ambiguous, rather than mere passive perception of ambiguous stimuli (Sun et al., 2017). It is worth noting that in the field of perceptual and cognitive neuroscience, different terms have been used to describe this event-related potential (ERP) component [e.g., P300, centro-parietal positive potential (CPP), and late positive deflection (LPD)], and the manipulation of attentional locus and stimulus-reward association drives this component (Hillyard et al., 1998; Mangun and Buck, 1998; Cravo et al., 2013; Itthipuripat et al., 2015), consistent with its role in coding stimulus ambiguity and task uncertainty.

Given the LPP’s diverse roles in coding faces, emotion, uncertainty, decisions, and combinations of these perceptual attributes, in this study, we propose that the LPP is a general neural signature of perceptual ambiguity, rather than a specific signature of emotional or affective responses. To test this hypothesis, we employed a range of experiments and investigated how stimulus-driven and task-driven factors modulate the LPP. Importantly, to investigate whether the LPP is a common neural substrate for various forms of perceptual ambiguity, we used either the same task or the same stimuli so that our results were comparable across experiments. First, using faces along two different morph dimensions as well as morphed animals, we showed that the LPP encoded perceptual ambiguity generally regardless of facial emotions or even faces. Second, using task instructions with different levels of ambiguity, we found that the LPP was modulated by task instructions and had the maximal response when the dimension of stimulus ambiguity was task relevant. Third, to specify the functional role of the LPP, we constructed regression models, which revealed that the LPP was specifically associated with response latency and confidence rating. Finally, we showed that our findings were further supported by direct behavioral ratings of task ambiguity and difficulty but could not be attributed to any differences in eye movements. Together, our findings show that the LPP encodes decisions under perceptual ambiguity in a general fashion, but is subject to whether the task dimension was relevant.

## Materials and Methods

### Subjects

In experiment 1 (face judgment task with fear-happy morphed emotions), 16 subjects (9 female, mean age ± SD, 20.1 ± 1.50 years) participated in the electroencephalogram (EEG) experiment, and 24 subjects (16 female, 22.3 ± 3.39 years) participated in the eye tracking experiment. Eleven subjects (9 female, 20.6 ± 2.80 years) participated in experiment 2 (face judgment task with anger-disgust morphed emotions) and experiment 3 (animal judgment task with cat-dog morphs). In addition, 16 subjects (11 female, 19.63 ± 0.96 years) participated in experiments 4–6 (face judgment task with fear-happy morphed emotions but different task instructions). All subjects provided written informed consent according to protocols approved by the institutional review board.

### Stimuli

**Fig. 1.**
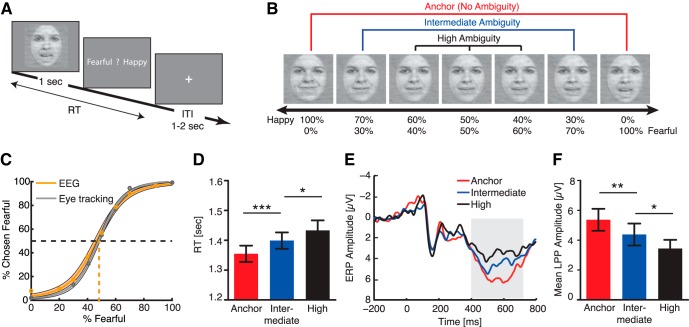
Experiment 1. ***A***, Task. A face was presented for 1 s followed by a question asking subjects to identify the facial emotion (fearful or happy). Faces are not shown to scale. ***B***, Sample stimuli of one female identity ranging from 0% fear/100% happy to 100% fear/0% happy. Three ambiguity levels (anchor, intermediate, and high) are grouped as shown above the stimuli. ***C***, Group average of psychometric curves showing the proportion of trials judged as fearful as a function of morph levels ranging from 0% fearful (100% happy; on the left) to 100% fearful (0% happy; on the right). Shaded area denotes ±SEM across subjects. ***D***, The reaction time (RT; relative to stimulus onset) for the fear/happy decision. Subjects judged facial emotions faster for anchor faces but slower for more ambiguous faces. Error bars denote one SEM across subjects. ***E***, ERP at the electrode Pz differentiated ambiguity levels. Gray shaded area denotes the LPP interval. ***F***, Mean LPP amplitude showed a parametric modulation by stimulus ambiguity. LPP amplitudes were averaged across the entire interval (shaded area in ***E***). Error bars denote one SEM across subjects. Paired *t* test between adjacent levels of ambiguity: *, *p* < 0.05; **, *p* < 0.01; and ***, *p* < 0.001.

The stimuli of experiment 1 (face judgment task with fear-happy morphed faces) were described in detail in a previous study (Wang et al., 2017). Briefly, stimuli were morphed expression continua between four exemplars (two female) of fearful and happy expressions as well as unambiguous anchor faces with clear fearful and happy expressions for each exemplar. We created 5 levels of fear-happy morphs, ranging from 30% fear/70% happy to 70% fear/30% happy in steps of 10% (Fig. 1*B*). Low-level image properties were equalized (Wang et al., 2017).

In experiment 2 (face judgment task with anger-disgust morphed emotions), anger-disgust morphs were created by FaceGen modeler (http://facegen.com /). Similar to fear-happy morphs, we also selected 4 identities [2 males and 2 females from 3D human face models; two Asian (1 male and 1 female) and two white], with 2 anchors and 5 morph levels for each identity. The morphs ranged from 30% anger/70% disgust to 70% anger/30% disgust in steps of 10%.

In experiment 3 (animal judgment task with cat-dog morphs), cat-dog morphed stimuli were used and described in detail in a previous study (Freedman et al., 2001). In brief, there were two cat identities and two dog identities. Each cat identity was morphed with another dog identity to create 4 morph lines. Similarly, each morph line had 2 anchors and 5 morph levels (20% cat/80% dog, 40% cat/60% dog, 50% cat/50% dog, 60% cat/40% dog, and 80% cat/20% dog). Experiments 4–6 (face judgment task using instructions with levels of ambiguity) used the identical stimuli as experiment 1, but with different task instructions (see below).

In experiment 1, there were 252 trials in 3 blocks for eye tracking subjects and 252 trials in 2 blocks for EEG subjects. In experiments 2 and 3, there were 252 trials in 2 blocks. In experiments 4–6, there were 280 trials in 2 blocks for each experiment. All trials were pooled for analysis.

### Task

In experiment 1, in each trial, a face was presented for 1 s followed by a question prompt asking subjects to make the best guess of the facial emotion. After stimulus offset, subjects had 2 s to respond, otherwise the trial was aborted and discarded. Subjects were instructed to respond as quickly as possible, but only after stimulus offset. No feedback message was displayed, and the order of faces was completely randomized for each subject. An intertrial interval (ITI) was jittered randomly with a uniform distribution of 1–2 s. In each block, there were equal numbers of trials for each morph level and each identity.

**Fig. 2.**
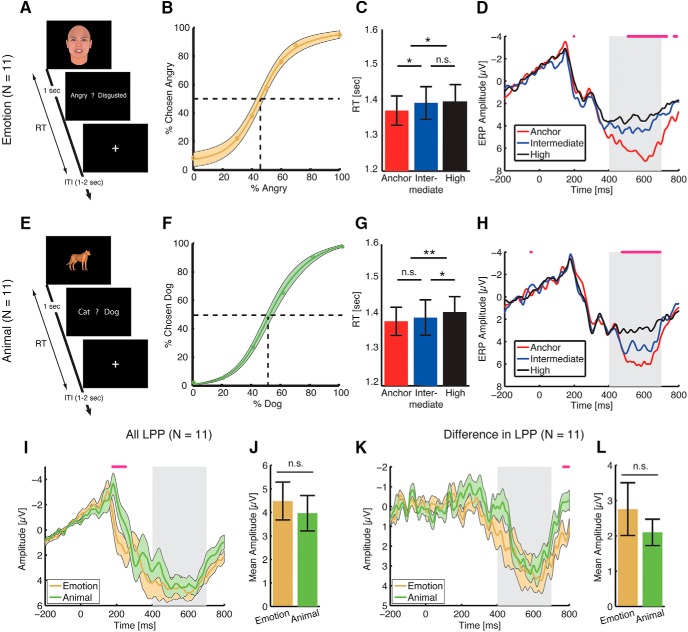
Experiments 2 and 3. ***A–D***, Experiment 2: face judgment task with anger-disgust morphed emotions. ***E–H***, Experiment 3: animal judgment task with cat-dog morphs. ***A***, ***E***, Task. A face (***A***) or an animal (***E***) was presented for 1 s followed by a question asking subjects to identify the facial emotion (angry or disgusted; ***A***) or animal category (cat or dog; ***E***). Faces and animals are not shown to scale. ***B***, ***F***, Group average of psychometric curves showing the proportion of trials judged as angry (***B***) or dog (***F***) as a function of morph levels. Shaded area denotes ±SEM across subjects. ***C***, ***G***, RT. Subjects judged unambiguous faces (***C***) or animals (***G***) faster than ambiguous faces or animals. Error bars denote one SEM across subjects. Paired *t* test between adjacent levels of ambiguity: *, *p* < 0.05; **, *p* < 0.01; n.s., not significant. ***D***, ***H***, ERP at the electrode Pz differentiated ambiguity levels. Both experiments showed a larger LPP for anchors and a smaller LPP for high ambiguity, consistent with the face judgment task with fear-happy morphed emotions. Gray shaded area denotes the LPP interval (400–700 ms after stimulus onset). The top magenta bars illustrate the points with significant difference across three ambiguity levels (one-way repeated-measure ANOVA, *p* < 0.05, corrected by false discovery rate for *Q* < 0.05). ***I***, ***J***, Comparison between experiments 2 and 3 on all LPP (average across all conditions). ***K***, ***L***, Comparison between experiments 2 and 3 on the difference in LPP (anchor minus high). ***I***, ***K***, ERP. Shaded areas denote ±SEM across subjects. Gray shaded area denotes the LPP interval. The top magenta bars illustrate the points with significant difference between the two tasks (paired *t* test, *p* < 0.05, corrected by false discovery rate for *Q* < 0.05). There was no significant difference in the LPP between the two tasks for both all LPP (***I***) and the difference in LPP (***K***), although the animal task had more negative ERP ∼200 ms for all LPP (***I***). ***J***, ***L***, Mean LPP amplitude. LPP amplitudes were averaged across the entire interval (400–700 ms after stimulus onset). Error bars denote one SEM across subjects. There was no significant difference between the two tasks for both all LPP (***J***; paired *t* test, *p* = 0.45) and the difference in LPP (***L***; *p* = 0.41).

Experiments 2 and 3 had the same task structure as experiment 1. In experiment 2 (Fig. 2*A*), subjects were asked to make the best guess of the facial emotion (anger or disgust), and in experiment 3 (Fig. 2*E*), subjects were asked to make the best guess of the animal category (cat or dog).

**Fig. 3.**
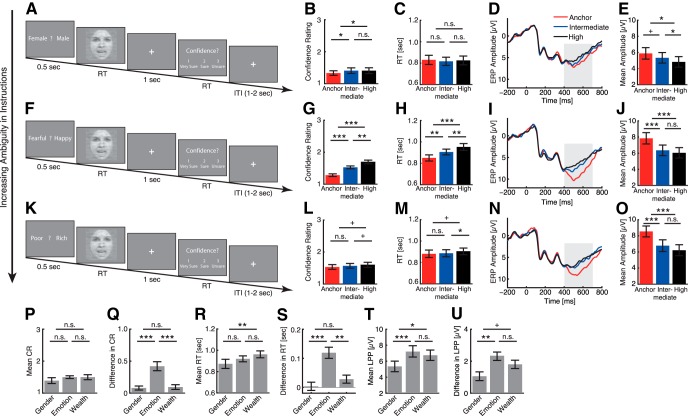
Experiments 4–6. ***A–E***, Experiment 4: gender judgment task. ***F–J***, Experiment 5: emotion judgment task. ***K–O***, Experiment 6: wealth judgment task. ***A***, ***F***, ***K***, Task. A question prompt was presented for 500 ms, followed by the stimulus. Subjects were instructed to respond as quickly as possible, and the stimulus stayed on the screen until subjects responded. ***B***, ***G***, ***L***, Confidence ratings (CR). Confidence ratings systematically varied as a function of stimulus ambiguity for the emotion judgment task but not for the gender judgment task nor wealth judgment task. ***C***, ***H***, ***M***, RT. RT can be considered as an implicit measure of confidence, and had a similar pattern as confidence ratings. ***D***, ***I***, ***N***, ERP at the electrode Pz. Gray shaded area denotes the LPP interval. ***E***, ***J***, ***O***, Mean LPP amplitude. Error bars denote one SEM across subjects. Paired *t* test between adjacent levels of ambiguity: +, *p* < 0.1; *, *p* < 0.05; **, *p* < 0.01; ***, *p* < 0.001; n.s.: not significant. ***P***, Mean confidence rating for each experiment. ***Q***, Difference in confidence rating between anchor and high-ambiguity stimuli for each experiment. ***R***, Mean RT for each experiment. ***S***, Difference in RT between anchor and high-ambiguity stimuli for each experiment. ***T***, Mean LPP averaged across all conditions for each experiment. ***U***, Difference in LPP between anchor and high-ambiguity stimuli for each experiment. Error bars denote one SEM across subjects. Paired *t* test between subjects: +, *p* < 0.1; *, *p* < 0.05; **, *p* < 0.01; ***, *p* < 0.001; n.s., not significant.

Experiments 4–6 also had a similar task structure as experiment 1. However, notably, experiments 4–6 were speeded tasks—a question prompt was presented for 500 ms, followed by the stimulus. Subjects were instructed to respond as quickly as possible, and the stimulus stayed on the screen until subjects responded. Subjects had 2 s to respond, otherwise the trial was aborted and discarded. No feedback message was displayed, and the order of stimuli was completely randomized for each subject. In experiment 4 (Fig. 3*A*), subjects were asked to judge the gender of the face. In experiment 5 (Fig. 3*F*), subjects were asked to make the best guess of the facial emotion (identical to experiment 1). In experiment 6 (Fig. 3*K*), subjects were asked to guess the wealth (poor versus rich) of the face model.

Eye-tracking subjects in experiment 1 and subjects in experiments 4–6 also performed a confidence rating. After emotion judgment and a 500-ms blank screen (1 s of fixation for experiments 4–6), subjects were asked to indicate their confidence of judgment, by pushing the button 1 for “very sure,” 2 for “sure,” or 3 for “unsure.” As with the emotion judgment, subjects had 2 s to respond before the trial was aborted, and no feedback message was displayed.

### Behavioral data analysis

We fitted a logistic function to obtain smooth psychometric curves:

$$P\left(x\right)=\frac{P_{\inf}}{1+e^{-\alpha \left(x-x_{half}\right)}},$$

where *P* is the percentage of trials judging faces as fearful in experiment 1, judging faces as angry in experiment 2, and judging animals as dog in experiment 3; *x* is the stimulus level; *P_inf_* is the value when *x* approaches infinity (the curve’s maximum value); *x_half_* is the symmetric inflection point (the curve’s midpoint); and α is the steepness of the curve. *P_inf_*, *x_half_*, and α were fitted from the observed data (*P* and *x*). We derived these parameters for each subject.

### Eye tracking and apparatus

Twenty-four subjects participated in the eye-tracking experiment. The stimuli were presented using Matlab with the Psychophysics Toolbox (Brainard, 1997). Fourteen healthy subjects were recorded with a head-supported noninvasive infrared EyeLink 1000 System (SR Research). One of the eyes was tracked at 1000 Hz. Subjects were seated at a distance of 60 cm in front of a computer screen in a dimly lit, sound-attenuated room. The experiment was administered on a 20-inch (40 × 30-cm screen size) Lenovo CRT display (1024 × 768 screen resolution). The eye tracker was calibrated with the built-in 9-point grid method at the beginning of each block. Fixation extraction was conducted using software supplied with the EyeLink eye tracking system. Saccade detection required a deflection of >0.1°, with a minimum velocity of 30°/s and a minimum acceleration of 8000°/s^2^. Fixations were defined as the complement of a saccade, i.e., periods without saccades, and the fixation locations were determined using the EyeLink event parser.

Ten healthy subjects were recorded using a remote noninvasive infrared Tobii T120 system which recorded binocular gaze at 120 Hz. The Tobii visualization software (Tobii Studio 2.2) was used to record eye movements and perform gaze analysis. Fixations were detected by Tobii Fixation Filter implemented in Tobii Studio. The Tobii Fixation Filter is a classification algorithm proposed by (Olsson, 2007) and detects quick changes in the gaze point using a sliding window averaging method. Velocity threshold was set to 35 pixels/sample, and distance threshold was set to 35 pixels in our study.

To quantitatively compare the fixation properties within certain parts of the face, we defined three regions of interest (ROIs): eyes, mouth, and center (Fig. 5*A*). Each ROI is a rectangle, and the eye and mouth ROI have the same size. To compute fixation density maps, fixation locations were smoothed with a 40-pixel 2D Gaussian kernel with a standard deviation of 10 pixels. The fixation density map indicates the probability of fixating a given location (in arbitrary units), which was calculated based on the number and duration of fixations and which ensured an equal contribution from each subject and statistical independence between subjects. The average fixation density within the ROIs was calculated for each subject and for each morph level during the entire 1-s stimulus period. Statistical comparisons were then performed to compare whether the mean fixation density, the total fixation duration, the mean fixation duration, the percentage of the number of fixations, and the latency of the first fixation falling into an ROI differed between ambiguity levels, for each ROI (Fig. 5*B–F*).

### EEG data recording

Subjects were seated comfortably ∼1.1 m in front of a computer screen in a dimly lit and electromagnetically shielded room. Experiments were administered on a 19-inch (37.7 × 30.1-cm screen size) IBM LCD display (1280 × 1024 screen resolution). Stimuli were presented using E-prime. EEGs were recorded using a digital AC amplifier from 32 scalp sites with tin electrodes mounted in an elastic cap (NeuroScan4.5) according to the International 10-20 system. EEGs were recorded from the following sites: frontal, FP1, FP2, F7, F3, Fz, F4, F8; frontal-central, FC3, FCz, FC4; central, C3, Cz, C4; central-parietal, CP3, CPz, CP4; parietal, P7, P3, Pz, P4, P8, frontal-temporal-parietal, FT7, TP7, T7, T8, TP8, FT8; and occipital, O1, Oz, O_2_. The vertical electro-oculograms (VEOGs) were recorded from left supra-orbital and infra-orbital electrodes. The horizontal electro-oculograms (HEOGs) were measured from electrodes placed lateral to the outer canthi of the left and right eyes. The ground electrode was placed on the forehead. One reference electrode was placed at the left mastoid and the other at the right mastoid, and all recordings were referenced to the right mastoid. All impedance was maintained at less than 5 ΚΩ. EEGs and electro-oculograms (EOGs) were amplified using a 0.05- to 70-Hz bandpass filter and were continuously sampled at 500 Hz/channel.

### EEG data preprocessing

EEG data were processed using EEGLAB (Delorme and Makeig, 2004), an open-source toolbox running in the Matlab environment, and in-house Matlab functions. The continuous EEG data were re-referenced to the average of the left and right external mastoid signals to avoid biasing the data toward one hemisphere (Nunez and Srinivasan, 2006; Luck, 2014). The data were filtered using a digital zero-phase shift bandpass filter of 0.5-30 Hz with a slope of 24 dB/octave. Then the continuous EEG data were epoched into 1-s segments (–200 to 800 ms relative to stimulus onset), and the prestimulus interval (–200 to 0 ms) was used as the baseline. We did not extend the epoch beyond 800 ms, as previous studies have suggested the termination of LPP effects at 800 ms (Schupp et al., 2000; Hu et al., 2014). The data were then baseline corrected by subtracting the average activity during the baseline period. Trials that had blinks in any part of the segment were excluded using a blink detection tool from ERPlab (http://erpinfo.org/erplab), in which vertical ocular artifacts exceeding a normalized cross-variance threshold of 0.7 were detected during the whole epoch (Lopez-Calderon and Luck, 2014; Luck, 2014). We rejected these trials because blinks might not only alter the sensory input of that trial, but also contaminate the EEG signals, especially the signals from the frontal channels. Epochs with saccadic eye movements were detected and discarded using a step-like artifact-detection function, in which horizontal ocular artifacts exceeding 70 μV in amplitude were detected during the entire epoch with 200-ms moving window and 50-ms increment steps. This function is suitable to detect saccadic eye movements that typically consist of sudden, step-like changes in voltage (Lopez-Calderon and Luck, 2014). Remaining artifacts were further detected using a moving-window peak-to-peak artifact-detection method on specific midline electrodes. Epochs were excluded if the peak-to-peak voltage (the difference between the largest and smallest values) exceeded a threshold of 100 μV. Bad channels were interpolated using the average voltage from their surrounding electrodes.

### EEG stimulus-locked ERP analysis

Within each subject, mean wave form of each morph level was computed, time-locked to the onset of the stimulus. Single-subject mean waveforms were subsequently averaged to obtain group-level mean waveforms. Here, we measured the LPP (entire wave form) based on the time window of 400 to 700 ms after stimulus onset at the parietal-central (Pz) electrode (Sabatinelli et al., 2007). Importantly, the scalp topography of the difference wave form between high ambiguity and anchor showed the most pronounced difference at Pz in this time window (Sun et al., 2017).

### Regression models

We constructed regression models to understand to what extent behavioral measurements could be explained by the LPP. We employed linear mixed models (LMM) for reaction times (RTs) and confidence rating. We used single-trial mean LPP amplitude (400–700 ms after stimulus onset) and stimulus ambiguity level as fixed effects, together with by-subject random intercept. Statistical significance of the model was computed by likelihood ratio tests of the full model with the fixed effect of LPP amplitude against a null model without the fixed effect of the LPP.

The formula for RT is

RT ∼ 1 + LPP + ambiguity level + (1 + 1 | subID).

The formula for confidence rating is

confidence rating ∼ 1 + LPP + ambiguity level + (1 + 1 | subID).

Because behavioral choice was binary, we employed a logistic regression model for behavioral choice. The formula for behavioral choice is

logit (choice) ∼ 1 + LPP + RT + confidence rating + subID; distribution = binomial.

Note that for behavioral choice, the contribution of RT and confidence rating was accounted to control for task difficulty.

## Results

What are the neural substrates underlying ambiguity processing? To answer this question, we conducted a series of EEG experiments with a variety of ambiguous stimuli. We not only analyzed the judgment choice behavior (i.e., the parametric relationship between stimulus level and behavioral response) to investigate the judgment sensitivity and specificity, but also used both explicit (confidence ratings) and implicit (RT) behavioral measures to index response to stimulus ambiguity and judgment difficulty. In particular, previous findings argue that a late positive potential (LPP) originating from the parietal-central (Pz) electrode is involved in affective valence (pleasant, unpleasant, and neutral) and arousal processing (Cuthbert et al., 2000; Schupp et al., 2000; Codispoti et al., 2007; Pastor et al., 2008; Leite et al., 2012). We here examined whether and how the LPP was modulated by stimulus ambiguity. In a later section, we directly linked stimulus ambiguity, behavioral indices of ambiguity (RT and confidence ratings), and the neural response (the LPP) through a regression model, which revealed a specific functional role of the LPP.

### Experiment 1: The LPP encodes perceptual ambiguity of fear-happy morphed faces

In this experiment, we asked subjects to judge emotional faces as fearful or happy (Fig. 1*A*). Faces were either unambiguously happy, unambiguously fearful (“anchors”), or graded ambiguous morphs between the two emotions (Fig. 1*B*). Because emotion ambiguity was symmetric between emotion intensity levels, we grouped the seven emotion levels into three ambiguity levels (Fig. 1*B*): anchor, intermediate ambiguity (30%/70% morph), and high ambiguity (40% to 60% morph). For each subject, we quantified behavior as the proportion of trials identified as fearful as a function of morph level (Fig. 1*B*). We found a monotonically increasing relationship between the likelihood of identifying a face as fearful and the fearfulness in the morphed face for both subject groups (Fig. 1*C*), showing that subjects could well track the gradual change in the emotions. There was also a systematic relationship between RT and ambiguity: the more ambiguous the stimulus, the longer the RT (Fig. 1*D*; one-way repeated-measure ANOVA of ambiguity levels, *F*(2,30) = 15.6, *p* = 2.27 × 10^−5^, η_p_^2^ = 0.51).

Confirming our previous finding (Sun et al., 2017), we found that the LPP component showed a parametric relationship with the degree of ambiguity shown in the stimuli, suggesting that LPP amplitude can index the level of perceptual ambiguity (Fig. 1*E*). Consistent with prior literature on the LPP (Pastor et al., 2008; Leite et al., 2012), we focused on the electrode Pz during the time interval of 400–700 ms after stimulus onset. Our results were confirmed by the mean LPP amplitude (Fig. 1*F*; one-way repeated-measure ANOVA of ambiguity levels, *F*(2,30) = 11.3, *p* = 2.20 × 10^−4^, η_p_^2^ = 0.43), and *post hoc t* tests revealed a significant difference between anchor (5.36 ± 2.94 µv, mean ± SD) and intermediate ambiguity (4.38 ± 2.95 µv; paired two-tailed *t* test, *t*(15) = 3.03, *p* = 0.008, Cohen’s *d* = 0.78), and a significant difference between intermediate and high ambiguity (3.44 ± 2.33 µv; *t*(15) = 2.79, *p* = 0.014, *d* = 0.72). Interestingly, the difference between anchor versus intermediate ambiguous faces (0.99 ± 1.30 µv) was not different from that between intermediate versus most ambiguous faces (0.93 ± 1.35 µv; paired *t* test: *t*(15) = 0.12, *p* = 0.91, *d* = 0.03), indicating a similar transition between ambiguity levels.

### Experiments 2 and 3: The LPP encodes general perceptual ambiguity

Does the LPP only encode ambiguity about faces or even only along the fear-happy dimension? To answer this question, we asked 11 healthy subjects to judge facial emotions along the anger-disgust dimension (experiment 2; Fig. 2*A*), and animal categories of cat-dog morphs (experiment 3; Fig. 2*E*). Similar to fear-happy morphs, in both experiments, subjects could well track the gradual change of morph levels (Fig. 2*B*, *F*), and RT was faster for less ambiguous stimuli for both emotion judgment of anger versus disgust (Fig. 2*C*; one-way repeated-measure ANOVA of ambiguity levels, *F*(2,20) = 4.78, *p* = 0.019, η_p_^2^ = 0.30) and animal judgment of cat versus dog (Fig. 2*G*; *F*(2,20) = 8.48, *p* = 0.009, η_p_^2^ = 0.37).

In both experiments, we not only found a clear LPP component in the interval of 400–700 ms after stimulus onset at the electrode Pz as the fear-happy morph task, but importantly, the LPP also differentiated the ambiguity levels (emotion task, 508–730 ms; animal task, 474–692 ms), still with anchor faces showing the most positive potential (Fig. 2*D*, *H*). This observation was confirmed by the mean LPP amplitude (Fig. 2*D*; emotion task: anchor, 6.03 ± 3.70 µV; intermediate, 4.09 ± 2.87 µV; high, 3.30 ± 1.94 µV; one-way repeated-measure ANOVA of ambiguity levels, *F*(2,20) = 10.3, *p* = 8.41 × 10^−4^, η_p_^2^ = 0.51; Fig. 2*H*; animal task: anchor, 4.84 ± 2.89 µV; intermediate, 4.22 ± 2.59 µV; high, 2.85 ± 2.40 µV; *F*(2,20) = 12.8, *p* = 2.62 × 10^−4^, η_p_^2^ = 0.56). Our results were further corroborated using the peak amplitude of the LPP.

We next compared experiments 2 and 3 to explore the possible difference in coding ambiguity. Both the mean LPP across all conditions (Fig. 2*I*, *J*) and the difference in LPP between anchor and high ambiguity (Fig. 2*K*, *L*) were remarkably similar (two-tailed paired *t* test; mean, *t*(10) = 0.79, *p* = 0.45, *d* = 0.25; difference, *t*(10) = 0.87, *p* = 0.41, *d* = 0.28), suggesting that the LPP could be elicited by different forms of perceptual ambiguity similarly, thus encoding perceptual ambiguity in a general fashion. Together, our results showed that the LPP encoded perceptual ambiguity in general regardless of facial emotions or even faces.

### Experiments 4–6: The LPP’s coding of ambiguity was modulated by task instructions

We have shown above that the LPP encodes general stimulus-driven perceptual ambiguity. How does the LPP encode ambiguity in task instructions? To answer this question, we next employed identical stimuli but asked different questions when judging the stimulus. Using fear-happy morphed emotions as in experiment 1, task instructions thus had three levels of ambiguity: the gender judgment task (experiment 4; Fig. 3*A*) had no ambiguity because all four face models had clear genders, whereas the wealth judgment task (experiment 6; Fig. 3*K*) had the highest ambiguity because whether the face model is poor or rich could not be told without any priors. The emotion judgment task (experiment 5; Fig. 3*F*) had an intermediate ambiguity, and it was a direct replication of experiment 1. Note that only the task instruction of the emotion judgment task was relevant to the dimension of ambiguity in stimulus. Sixteen subjects participated in all three experiments, and the order of experiments was counterbalanced across subjects.

Behaviorally, after judging the stimulus, we asked subjects to report their confidence in their decisions. In the emotion judgment task, subjects reported significantly higher levels of confidence for anchor faces compared to ambiguous faces (Fig. 3*G*; one-way repeated-measure ANOVA of ambiguity levels: *F*(2,30) = 27.8, *p* = 1.48 × 10^−7^, η_p_^2^ = 0.65). However, in both gender judgment task (Fig. 3*B*; *F*(2,30) = 4.62, *p* = 0.018, η_p_^2^ = 0.24) and wealth judgment task (Fig. 3*L*; *F*(2,30) = 4.95, *p* = 0.014, η_p_^2^ = 0.25), although subjects still reported different levels of confidence for different ambiguity levels, confidence did not decrease systematically as a function of increasing stimulus ambiguity, and the difference in confidence between ambiguity levels was much smaller. In addition to the explicit confidence ratings, RT can be considered as an implicit measure of confidence. In the emotion judgment task, RT was faster for anchor faces compared to ambiguous faces (Fig. 3*H*; *F*(2,30) = 20.8, *p* = 2.15 × 10^−6^, η_p_^2^ = 0.58), replicating the results in experiment 1 (Fig. 1*D*). Similarly, RT mirrored confidence ratings in both gender judgment task (Fig. 3*C*; *F*(2,30) = 0.28, *p* = 0.76, η_p_^2^ = 0.018) and wealth judgment task (Fig. 3*M*; *F*(2,30) = 3.42, *p* = 0.046, η_p_^2^ = 0.19). Together, our results show that stimulus-driven ambiguity modulates behavior to a much weaker extent when it is not task relevant.

We further compared across experiments. Although the mean confidence rating averaged cross all stimuli was similar across experiments (Fig. 3*P*; one-way repeated-measure ANOVA of experiments: *F*(2,30) = 1.07, *p* = 0.36, η_p_^2^ = 0.067), the mean RT was significantly longer in the wealth judgment task (Fig. 3*R*; *F*(2,30) = 3.70, *p* = 0.037, η_p_^2^ = 0.20), whose task instruction had the highest level of ambiguity, thus confirming the manipulation task ambiguity. Furthermore, the difference in confidence ratings between the high-ambiguity faces and anchor faces was significantly greater in the emotion judgment task compared with the gender and wealth judgment tasks (Fig. 3*Q*; *F*(2,30) = 17.4, *p* = 9.62 × 10^−6^, η_p_^2^ = 0.54), and the difference in RT was also significantly greater in the emotion judgment task compared with the gender and wealth judgment tasks (Fig. 3*S*; *F*(2,30) = 18.2, *p* = 6.67 × 10^−6^, η_p_^2^ = 0.55). However, the difference in both confidence and RT was similar between the gender judgment task and wealth judgment task (Fig. 3*Q*, *S*; both *p* > 0.05), suggesting that task-driven ambiguity did not simply add or multiply to the stimulus-driven ambiguity. Furthermore, when subtracting the RT in the emotion judgment task from the RT in the gender judgment task at each ambiguity level, we still observed that the RT increased as a function of increasing stimulus ambiguity (anchor, 4.96 ± 175.8 ms; intermediate, 65.3 ± 146.4 ms; high, 123.6 ± 177.8 ms; one-way repeated-measure ANOVA of ambiguity levels, *F*(2,30) = 17.8, *p* < 0.001, η_p_^2^ = 0.51), suggesting that behavioral response was modulated by stimulus ambiguity more strongly when the task instruction was relevant. Together, both explicit confidence ratings and implicit confidence measures by RT suggested that stimulus-driven ambiguity modulated behavior maximally if it matched the task instruction.

Neurally, we first showed that experiment 5 replicated the findings in experiment 1 (Fig. 3*I*), although experiment 5 was a speeded task. We also observed LPP effects in both gender judgment task (Fig. 3*D*) and wealth judgment task (Fig. 3*N*). However, the LPP in the gender judgment task had a lower amplitude (Fig. 3*D*), although the gender judgment task had a lower ambiguity in task instruction compared to the emotion judgment task, a result opposite to LPP’s coding of stimulus-driven ambiguity. Furthermore, although the LPP could also differentiate levels of stimulus ambiguity in the gender judgment task (Fig. 3*E*; *F*(2,30) = 8.48, *p* = 0.0012, η_p_^2^ = 0.36), the coding of stimulus ambiguity was relatively weaker compared with the emotion judgment task (Fig. 3*J*; *F*(2,30) = 48.0, *p* = 4.48 × 10^−10^, η_p_^2^ = 0.76) and the wealth judgment task (Fig. 3*O*; *F*(2,30) = 33.3, *p* = 2.41 × 10^−8^, η_p_^2^ = 0.69; also see below), suggesting that the LPP encoded stimulus ambiguity more strongly when the task involved ambiguity in judgment.

We further confirmed our findings by comparing across experiments: the overall LPP averaged cross all stimuli varied across experiments even with the identical stimuli (Fig. 3*T*; one-way repeated-measure ANOVA of experiments: *F*(2,30) = 9.83, *p* = 5.21 × 10^−4^, η_p_^2^ = 0.40), with the emotion and wealth judgment tasks featuring a larger mean LPP. Similarly, the difference in LPP between the anchor faces and high-ambiguity faces was significantly greater in the emotion and wealth judgment tasks compared with the gender judgment task (Fig. 3*U*; *F*(2,30) = 6.05, *p* = 0.0062, η_p_^2^ = 0.29). Although there were levels of ambiguity in top-down instructions, the LPP had the maximal response to stimulus ambiguity when the dimension of stimulus ambiguity matched the task instruction, instead of when task instruction had the highest or lowest ambiguity. It is worth noting that in contrast to the coding of stimulus ambiguity where the LPP amplitude was largest for anchors (Fig. 3*I*, *J*), the most ambiguous wealth judgment task elicited both a greater overall LPP (Fig. 3*T*; paired *t* test: *t*(15) = −2.56, *p* = 0.022, *d* = −0.66) and a greater difference in LPP (Fig. 3*U*; paired *t* test: *t*(15) = −1.95, *p* = 0.071, *d* = −0.50) compared with the least ambiguous gender judgment task. This indicated that the LPP was modulated by task instruction differently compared to stimulus.

Finally, it is worth noting that in these three experiments we varied task ambiguity but kept the stimuli identical, and there might be an interaction between task ambiguity and stimulus ambiguity. The stimulus ambiguity might explain some of the LPP effects. For example, in the gender judgment task, although the ambiguity in task was minimal, some stimuli were still ambiguous in emotions, which might drive the LPP’s response (Fig. 3*D*, *E*). Therefore, our data indicated that even if subjects were not judging emotions, the emotion ambiguity might still be encoded to some extent. It is also notable that behavioral choice was not correlated with ambiguity levels for any of the tasks (gender, *r* = 0.046, *p* = 0.63; emotion, *r* = 0.023, *p* = 0.81; wealth, *r* = *−*0.033, *p* = 0.73), ruling out the possibility that the LPP could be simply explained by behavioral choices.

Taken together, our neural data suggested that the LPP was modulated by task instructions and encoded stimulus ambiguity more strongly when the task instruction involved ambiguity. This result has shown context dependency of the LPP and is consistent with our previous finding that the LPP is generated only when decisions are made on a dimension that is ambiguous (Sun et al., 2017).

### Regression models revealed a trial-by-trial coupling between the LPP and behavioral measurements

We have shown that the LPP encodes general stimulus-driven perceptual ambiguity and is modulated by task-driven ambiguity. What is the specific functional role of the LPP when it encodes perceptual ambiguity? Is it associated with perceptual representation of the stimulus or with making judgments about the stimulus? We next conducted regression analyses (see Methods) to answer these questions.

First, we analyzed the relationship between the LPP and RT, accounting for the contribution from stimulus ambiguity. In the emotion judgment task (experiment 5), we found that in the full model, LPP amplitude could predict RT with a significant regression coefficient (slope; β = −4.72, *p* < 0.001), and similarly for ambiguity level (β = 66.28, *p* < 0.001) and intercept (β = 855.5, *p* < 0.001). Importantly, the full model with the fixed effect of LPP significantly outperformed the null model (χ^2^(5) = 57.69, *p* < 0.001). These results suggested that the variance of RT could be well explained by the variance of LPP amplitude, even when the contribution of stimulus ambiguity was regressed out. Notably, similar results were found for the anger-disgust judgment (experiment 2; χ^2^(5) = 5.06, *p* = 0.024) and cat-dog judgment (experiment 3; χ^2^(5) = 5.46, *p* = 0.019) tasks, confirming the generality of the LPP in coding perceptual ambiguity. We also found that the RT could be explained by the LPP in the gender judgment (experiment 4; χ^2^(5) = 40.1, *p* < 0.001) and wealth judgment (experiment 6; χ^2^(5) = 32.2, *p* < 0.001) tasks.

Second, we built a regression model to explain the variance in confidence rating. In the emotion judgment task, we found that the LPP amplitude could well predict confidence rating (χ^2^(5) = 25.6, *p* < 0.001), even when the contribution of stimulus ambiguity was regressed out. Similar results were also found for the gender judgment (χ^2^(5) = 7.65, *p* = 0.005) and wealth judgment (χ^2^(5) = 5.63, *p* = 0.017) tasks.

Finally, we built a logistic regression model to predict behavioral choice (e.g., judgments of fear or happy in experiment 1). We found that the LPP amplitude could not predict behavioral choice for the emotion judgment task (β = 0.0009, *p* = 0.82). Consistent with the generality of the LPP in coding stimulus-driven ambiguity, we found that the LPP could not predict behavioral choice for the anger-disgust judgment task (β = 0.0074, *p* = 0.23) or cat-dog judgment task (β = 0.0071, *p* = 0.26) either. However, the LPP could predict behavioral choice for the wealth judgment task (β = 0.014, *p* < 0.001), but not gender judgment task (β = −0.0017, *p* = 0.68).

Altogether, we found that independent of stimulus ambiguity, the LPP was strongly coupled with response latency and confidence, two variables directly associated with perceptual ambiguity, but not behavioral choice, which was not directly related to perceptual ambiguity. Our results have therefore revealed a functional role of the LPP: it encodes decisions under perceptual ambiguity.

### Direct behavioral ratings of stimulus ambiguity and judgment difficulty confirmed our results

**Fig. 4.**
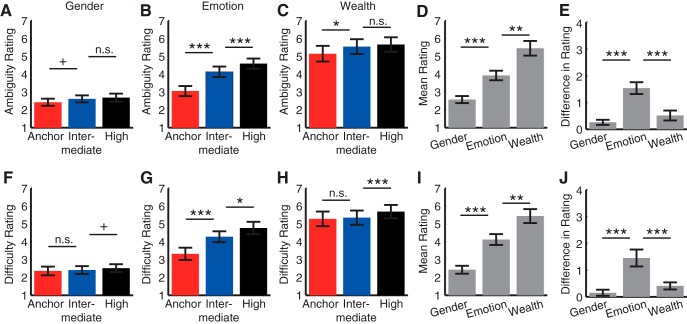
Direct behavioral ratings of stimulus ambiguity and judgment difficulty. ***A–E***, Ratings of stimulus ambiguity. Twenty-one raters rated the ambiguity of stimulus on a 1–10 scale. ***A***, Gender judgment task. For each stimulus, raters were asked, “how ambiguous is it to judge the gender of this face model?” We first averaged across stimuli for each ambiguity level within each subject and then averaged across subjects. Error bars denote one SEM across raters. ***B***, Emotion judgment task. Raters were asked, “how ambiguous is it to judge the emotion of this face model?” ***C***, Wealth judgment task. Raters were asked, “how ambiguous is it to judge the wealth of this face model?” ***D***, Mean ratings across tasks. ***E***, Difference in ratings between anchor and high-ambiguity stimuli. ***F–J***, Ratings of judgment difficulty. The same raters rated the judgment difficulty on a 1–10 scale. ***F***, Gender judgment task. Raters were asked, “how difficult is it to judge the gender of this face model?” ***G***, Emotion judgment task. Raters were asked, “how difficult is it to judge the emotion of this face model?” ***H***, Wealth judgment task. Raters were asked, “how difficult is it to judge the wealth of this face model?” ***I***, Mean ratings across tasks. ***J***, Difference in ratings between anchor and high ambiguity stimuli. Paired *t* test between adjacent levels of ambiguity: +, *p* < 0.1; *, *p* < 0.05; **, *p* < 0.01; ***, *p* < 0.001; n.s., not significant.

The levels of subjective ambiguity in stimulus and task in the above results were inferred from behavioral judgments, RTs, and confidence ratings. To further confirm our results, we next directly acquired subjective ratings for stimulus-driven ambiguity under each task instruction (see Fig. 4 legend for each rating question). Twenty-one subjects (16 female, 19.7 ± 1.59 years) rated the ambiguity of stimulus from the emotion judgment task (experiments 1 and 5) on a 1–10 scale. Indeed, anchor faces were rated the least ambiguous, whereas faces of high ambiguity were rated the most ambiguous (Fig. 4*B*; anchor, 3.05 ± 1.31; intermediate, 4.14 ± 1.34; high, 4.59 ± 1.30; one-way repeated-measure ANOVA of ambiguity levels, *F*(2,40) = 40.8, *p* < 0.001, η_p_^2^ = 0.67). Furthermore, the same subjects also rated the ambiguity in judging each stimulus for the gender judgment task (Fig. 4*A*; *F*(2,40) = 4.68, *p* = 0.015, η_p_^2^ = 0.19) and wealth judgment task (Fig. 4*C*; *F*(2,40) = 6.30, *p* = 0.01, η_p_^2^ = 0.24). Overall, the gender judgment task was rated the least ambiguous, whereas the wealth judgment task was rated the most ambiguous (Fig. 4*D*; gender, 2.57 ± 0.91; emotion, 3.93 ± 1.23; wealth, 5.45 ± 1.88; *p* = 1.63 × 10^−7^). Notably, compared with the emotion judgment task (Fig. 4*B*), the difference between ambiguity levels (Fig. 4*E*) was smaller in the gender judgment task (Fig. 4*A*) and the wealth judgment task (Fig. 4*C*), suggesting that subjects were less sensitive to stimulus ambiguity in these two tasks.

In addition, we asked the same subjects to rate the difficulty in judging the stimulus on a 1–10 scale, which mirrored the ambiguity ratings (Fig. 4*F–J*): faces of high ambiguity were rated the most difficult to judge (Fig. 4*G*; anchor, 3.32 ± 1.58; intermediate, 4.28 ± 1.40; high, 4.77 ± 1.59; one-way repeated-measure ANOVA of ambiguity levels, *F*(2,40) = 18.5, *p* < 0.001, η_p_^2^ = 0.48), and the wealth judgment task was rated the most difficult to perform (Fig. 4*I*; gender, 2.43 ± 1.02; emotion, 4.13 ± 1.39; wealth, 5.45 ± 1.74; *p* < 0.001). Again, the difference in rating between ambiguity levels (Fig. 4*J*) was smaller in the gender judgment (Fig. 4*F*; *F*(2,40) = 1.39, *p* = 0.26, η_p_^2^ = 0.065) and wealth judgment (Fig. 4*H*; *F*(2,40) = 8.11, *p* = 0.003, η_p_^2^ = 0.30) tasks.

Finally, we found that the ratings between stimulus ambiguity and judgment difficulty were highly correlated (gender, *r* = 0.75, *p* < 0.001; emotion, *r* = 0.86, *p* < 0.001; wealth, *r* = 0.92, *p* < 0.001), confirming that the more ambiguous stimuli were more difficult to judge (see Discussion).

In conclusion, direct behavioral ratings confirmed that our stimuli showed the anticipated subjective ambiguity, supporting the above behavioral and neurophysiological results.

### Eye movement analysis did not reveal difference across ambiguity levels

**Fig. 5.**
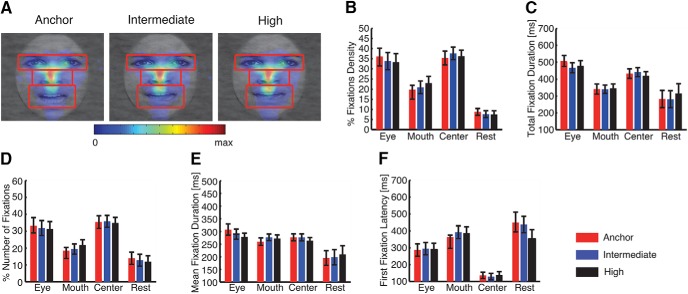
Eye movement comparisons across ambiguity levels in experiment 1. ***A***, Fixation density maps to quantify eye movements for each ambiguity level. Each map shows the probability of fixating a given location within a 1-s period after stimulus onset. The scale bar (color bar) is common for all plots (arbitrary units). The ROIs (eye, mouth, center) used for analysis are shown in red (not shown to subjects). ***B***, Percentage of fixation density in each ROI. ***C***, Total fixation duration in each ROI. ***D***, Percentage of the number of fixations in each ROI. ***E***, Average fixation duration in each ROI. ***F***, Latency of the first fixation onto each ROI. Error bars denote one SEM across subjects.

Could perceptual ambiguity lead to different fixation patterns on faces? To answer this question, we conducted an eye-tracking study with 24 subjects in experiment 1. We found indistinguishable fixation densities across ambiguity levels (Fig. 5*A*), in which subjects were equally likely to fixate the eye (Fig. 5*B*; one-way repeated-measure ANOVA of ambiguity levels, *p* = 0.91), mouth (*p* = 0.62), and center ROIs (*p* = 0.95), suggesting that subjects viewed faces similarly regardless of the ambiguity in faces. Furthermore, in each ROI, we found remarkably similar total fixation duration (Fig. 5*C*), percentage of the number of fixations (Fig. 5*D*), mean fixation duration (Fig. 5*E*), as well as latency to first fixate onto an ROI (Fig. 5*F*), across ambiguity levels (all *p* > 0.05). In conclusion, our eye tracking results showed that perceptual ambiguity did not bias eye movements, an important issue to consider, since it is well known that EEG data are prominently contaminated by potentials arising from the extraocular muscles. The similar strategy in viewing faces with different levels of ambiguity was expected, because our faces were presented briefly and preceded by a central fixation cross; however, these results argued that our behavioral and neurophysiological findings could not be attributed to differences in eye movements.

## Discussion

In this study, we investigated how the LPP was modulated by different types of perceptual stimuli and levels of ambiguity in task instructions. We found that the LPP encoded perceptual ambiguity of not only faces with different types morphed emotions, but also morphed animals, suggesting that the LPP encodes general perceptual ambiguity. On the other hand, using levels of ambiguity in task instructions, we found that the LPP was modulated by task instructions and had the maximal response when the dimension of stimulus ambiguity matched the task instruction. We further elucidated the relationship between stimulus ambiguity, behavioral response, and the LPP, and we found that the LPP was specifically associated with response latency and confidence rating. Finally, direct behavioral ratings of stimulus and task ambiguity confirmed our results, and we showed that our neurophysiological findings could not be explained by differences in eye movements. Taken together, our findings show that the LPP encodes decisions under perceptual ambiguity in a general fashion, but is subject to whether the task involves ambiguity in judgment.

A large literature shows that the LPP is modulated by emotional intensity and arousal and thus reflects an enhanced perception of emotional stimuli (Cuthbert et al., 2000; Schupp et al., 2000; Sabatinelli et al., 2007; Leite et al., 2012). However, recent studies show that the LPP encodes decisions by integrating sensory evidence and determining actions through a boundary-crossing criterion, similarly across multiple different tasks (O’Connell et al., 2012; Kelly and O’Connell, 2013; Murphy et al., 2015). Our previous findings have provided further specificity and mechanistic insight: the LPP not only differentiates levels of ambiguity, but is specifically associated with behavioral responses to ambiguous stimuli (not mere perception of ambiguous stimuli) and emerges only in the context of ambiguous stimuli (not when unambiguous stimuli are present alone; Sun et al., 2017). This prior study also suggested that the LPP originates from multiple loci in cingulate cortex using source modeling and functional MRI–guided ERP source prediction.

In the present study, we further established the generality of the LPP: the LPP encodes perceptual ambiguity of not only faces, but also animals. Our findings are consistent with several studies showing that the LPP can differentiate racially ambiguous faces (Willadsen-Jensen and Ito, 2006), ambiguous smiles (Calvo et al., 2013), and inconsistency of traits (Cacioppo et al., 1994). In particular, consistent with the present study, the LPP exhibits the same decision-predictive dynamics regardless of sensory modality (e.g., visual and auditory) and stimulus features (O’Connell et al., 2012), and it processes both real and fictive feedback in an instrumental learning task (Fischer and Ullsperger, 2013). Importantly, our results extended to affective stimuli beyond the simple sensory stimuli and suggested a general neural signature for perceptual ambiguity. A limitation of the present study is that all our perceptual decisions are still relatively simple; future studies will be needed to investigate whether our results can be extended to more complex decisions. Although our current results only speak to the ambiguity in the perceptual domain, it is an important future direction to investigate whether the same neural signature can encode ambiguity in economic decision-making, moral judgment, as well as other more complex settings.

It is worth noting that the terminology “ambiguity” in the perceptual domain refers to the categorical ambiguity (i.e., the uncertainty) of a stimulus belonging to one of two categories, with no missing information of a stimulus [see Sterzer and Kleinschmidt (2007) for a classic example of perceptual ambiguity that shares the same meaning of ambiguity as ours], whereas the term “ambiguity” in economic decision-making refers to the situation where the probability distribution itself is unknown (under risk, the probabilities of different outcomes can be estimated; whereas under ambiguity, even these probabilities are not known). Although it has been argued that individuals’ preferences for risk and ambiguity in economic decision-making are associated with different neural substrates, i.e., decision-making under ambiguity does not represent a special, more complex case of risky decision-making (Huettel et al., 2006; Krain et al., 2006), the LPP elicited in risky conditions (probabilities available) is found to be significantly greater than that in ambiguous conditions (probabilities unknown; Wang et al., 2015).

Judging perceptually ambiguous stimuli involves multiple processes, including but not limited to stimulus representation, decision-making, and motor output, and it is naturally associated with task difficulty, attention, conflict detection, and mental effort to resolve conflicts. To further dissociate these cognitive processes and provide a more specific functional role of the LPP, we conducted single-trial correlation analyses and regression analyses. We found that the LPP was strongly coupled with response latency and confidence and could well predict response latency and confidence. Notably, this was the case even when stimulus ambiguity was regressed out, suggesting that the LPP was associated with decisions but not stimulus representation. This is in line with previous reports (O’Connell et al., 2012; Sun et al., 2017), but we here extended the findings using a variety of perceptually ambiguous stimuli. Therefore, our present results have provided not only a further link between stimulus ambiguity, behavioral response, and the LPP, but also a more specific functional role of the LPP: this general ambiguity signature reflects decisions and responses when encoding various perceptual ambiguity. This ambiguity signature may thus index the difficulty in forming judgment and the mental effort to resolve conflict, but not merely representation of stimulus ambiguity or conflict detection. Moreover, this ambiguity signature may play an important role in generating the RT and confidence rating as we observed, consistent the origin of the LPP from the cingulate cortex (Yoder and Decety, 2014; Sun et al., 2017), which plays a critical role in cognitive control, detecting performance errors, and conflict monitoring (Cole et al., 2009; Alexander and Brown, 2010; Shackman et al., 2011; Sheth et al., 2012; Shenhav et al., 2013). Finally, ambiguity and confidence are two closely related variables that signal meta-information about decisions: ambiguity is based on the objective discriminability of the stimulus whereas confidence is based on the subjective judgment of the discriminability. Therefore, it is likely that the LPP is a general ambiguity signal that provides the underlying information necessary to judge the confidence in decisions, consistent with the idea that confidence judgment is a direct consequence of assessment of uncertainty (Kepecs and Mainen, 2012).

It is worth noting that task difficulty, attention, and RT are all intercorrelated to some extent, and examining unease/anxiety caused by ambiguity in decisions will be a clear future direction. However, our data argued against a simple role of attention because the stimulus should be equally attended when asking for a judgment of it; in particular, we observed similar results for a variety of stimuli across experiments. It has been shown that a larger LPP is associated with a shorter RT and thus an easier task, suggesting that the LPP reflects task difficulty (Kelly and O’Connell, 2013). When stimulus ambiguity was task relevant, we observed a similar coupling between the LPP and RT, which in turn indicated that the LPP reflected decision conflicts and mental effort to resolve such conflicts. Notably, however, our experiments using identical stimuli but task instructions with different levels of ambiguity showed that although RT became longer with increasing task difficulty (Fig. 3*R*; also see direct ratings of task difficulty in Fig. 4*E–H*), the LPP did not decrease as a function of increasing task difficulty (Fig. 3*T*, *U*), suggesting that the LPP does not play a simple role in encoding task difficulty associated with ambiguity and attention thereof.

One important aspect of the present study is to investigate context dependence of the LPP in coding perceptual ambiguity. We showed that the LPP was modulated by task instructions and responded more strongly when the task was ambiguous in nature, demonstrating the dependence of the LPP on the context (e.g., task to perform) and the interaction between stimulus ambiguity and task ambiguity. This is consistent with our previous finding that the LPP is generated only when the decisions involve choices about perceptual categories that are ambiguous, but not for choices involving the same stimuli but on a dimension that is unambiguous (e.g., judging whether the stimulus in experiments 2 and 3 is a face or animal; Sun et al., 2017). Furthermore, as shown above, using task instructions with different levels of ambiguity revealed a dissociation between the LPP’s role in coding stimulus-driven and task-driven ambiguity and task difficulty, and the coupling between the LPP and RT was also dependent on task instructions.

Uncertainty is critical in how stimulus information guides choice, and optimal decision-making requires continuous processing of ambiguity and uncertainty. Primate electrophysiology and human functional neuroimaging have revealed a distributed neural network encoding ambiguity and uncertainty, including the amygdala (Brand et al., 2007; Herry et al., 2007; Belova et al., 2008; Bermudez and Schultz, 2010; Wang et al., 2017), dopamine neurons of the ventral midbrain (Fiorillo et al., 2003), and the medial prefrontal cortex (Jenkins and Mitchell, 2010; Levy et al., 2010). In particular, prior studies have suggested that the LPP arises from brain regions including the dACC, vACC, PCC, and insula (Liu et al., 2012; Peng et al., 2012; Yoder and Decety, 2014; Sun et al., 2017; also see Weinberg et al., 2013). Human neuroimaging studies further supported these regions in ambiguity coding: ambiguous emotional faces relative to unambiguous emotional faces activate regions including the dACC, dlPFC, and IPL (Simmons et al., 2006); contrast between ambiguous and clear facial expressions induces activation in the dACC, dmPFC and IFG (Nomura et al., 2003); the vACC integrates confidence in judgment (De Martino et al., 2013; Lebreton et al., 2015); and intolerance of emotion ambiguity correlates with insula activation (Simmons et al., 2008). Together, these findings suggest that our brain has specific neural systems that process ambiguity and uncertainty. Our present results further reveal a common neural signature that encodes the general perceptual ambiguity and uncertainty and represent an important first step toward a neural explanation for complex human perceptual decision-making.

## Synthesis

Reviewing Editor: Philippe Tobler, University of Zurich Decisions are customarily a result of the Reviewing Editor and the peer reviewers coming together and discussing their recommendations until a consensus is reached. When revisions are invited, a fact-based synthesis statement explaining their decision and outlining what is needed to prepare a revision will be listed below. The following reviewer(s) agreed to reveal their identity: Marcus Grueschow, Michael Grubb.

The reviewers appreciated the removal of the risk task and the acquisition of additional data. However, one major interpretation issue remains: do the findings reflect a relation to ambiguity or task difficulty? With regard to this point, the reviewers felt that in its current form the paper has the potential to confuse more than to clarify. This issue should be addressed thoroughly.

Major issue

A primary source of confusion is the fact that the authors aim at identifying behaviorally relevant signatures of ambiguity but via several analyses convey a picture that seems to directly contradict this interpretation. For instance the authors state that: ‘These results suggested that the variance of RT could be well explained by the variance of LPP amplitude, even when the contribution of stimulus ambiguity was regressed out.’ Doesn't that mean that LPP amplitude reflects RT/task difficulty and is unaffected by ambiguity, while the main message of the paper is that LPP is directly linked to ambiguity?

More generally, it is unclear why the paper focuses on ambiguity rather than task difficulty directly. Also, it is unclear how the used operationalization of ambiguity relates to concepts such as conflict detection and/or conflict resolution. These concepts are not remotely touched upon anywhere. What exactly does an ambiguity signature reflect? What is it used for? Does it simply detect stimulus conflict or does it reflect its resolution? Which underlying mechanisms may generate the observed RTs and/or confidence ratings? What is the precise reason to introduce task-driven ambiguity if it is not differentiated from corresponding stimulus-driven ambiguity?

Other points

It is absolutely necessary to add formulas for the regressions used.

As initially suggested, logistic regressions on CHOICE not RT or confidence are necessary, as this is the behavior during which LPP is measured.

Wording issues

In: “We first used single-trial mean LPP amplitude (400 to 700ms after stimulus onset) and stimulus ambiguity level as the fixed effects, and by-subject random intercept as well as by-subject random slopes as random effects” please remove “as random effects” at the end.

In: “Furthermore, when stimulus ambiguity was regressed out, the LPP could still well predict behavioral responses, suggesting an independent role of the LPP in predicting behavioral responses” please specify what you mean with behavioral responses and keep in mind the major issue raised above.

“Taken together, our neural data suggested that although there were levels of ambiguity in task instructions and an LPP could be elicited by all three tasks, the LPP encoded stimulus ambiguity more strongly when the task instruction involved ambiguity, but not as a function of task ambiguity” is not understandable. Please reword.
